# Comparison of Amino Acid PET to Advanced and Emerging MRI Techniques for Neurooncology Imaging: A Systematic Review of the Recent Studies

**DOI:** 10.1155/2021/8874078

**Published:** 2021-01-20

**Authors:** Brittany M. Stopa, Csaba Juhász, Sandeep Mittal

**Affiliations:** ^1^Virginia Tech Carilion School of Medicine, Roanoke, VA, USA; ^2^Fralin Biomedical Research Institute, Roanoke, VA, USA; ^3^Departments of Pediatrics, Neurology, Neurosurgery, Wayne State University School of Medicine, Detroit, MI, USA; ^4^PET Center and Translational Imaging Laboratory, Children's Hospital of Michigan, The Karmanos Cancer Institute, Detroit, MI, USA; ^5^Carilion Clinic Neurosurgery, Roanoke, VA, USA

## Abstract

**Introduction:**

Standard neuroimaging protocols for brain tumors have well-known limitations. The clinical use of additional modalities including amino acid PET (aaPET) and advanced MRI (aMRI) techniques (including DWI, PWI, and MRS) is emerging in response to the need for more accurate detection of brain tumors. In this systematic review of the past 2 years of the literature, we discuss the most recent studies that directly compare or combine aaPET and aMRI for brain tumor imaging.

**Methods:**

A PubMed search was conducted for human studies incorporating both aaPET and aMRI and published between July 2018 and August 2020.

**Results:**

A total of 22 studies were found in the study period. Recent studies of aaPET with DWI showed a superiority of MET, FET, FDOPA, and AMT PET for detecting tumor, predicting recurrence, diagnosing progression, and predicting survival. Combining modalities further improved performance. Comparisons of aaPET with PWI showed mixed results about spatial correlation. However, both modalities were able to detect high-grade tumors, identify tumor recurrence, differentiate recurrence from treatment effects, and predict survival. aaPET performed better on these measures than PWI, but when combined, they had the strongest results. Studies of aaPET with MRS demonstrated that both modalities have diagnostic potential but MET PET and FDOPA PET performed better than MRS. MRS suffered from some data quality issues that limited analysis in two studies, and, in one study that combined modalities, overall performance actually decreased. Four recent studies compared aaPET with emerging MRI approaches (such as CEST imaging, MR fingerprinting, and SISTINA), but the initial results remain inconclusive.

**Conclusions:**

aaPET outperformed the aMRI imaging techniques in most recent studies. DWI and PWI added meaningful complementary data, and the combination of aaPET with aMRI yielded the best results in most studies.

## 1. Introduction

The standard-of-care neuroimaging modality for detection of brain tumors is contrast-enhanced magnetic resonance imaging (CE-MRI), which is a critical component of the clinical management strategy, from diagnosis to prognosis as well as treatment response assessment [1]. However, standard MRI has some important clinical limitations [2]. It cannot be relied upon for definitive diagnosis or tumor grading; therefore, pathology remains the diagnostic benchmark. Conventional MRI has limited utility for predicting prognosis or outcomes, and it has limited correlations with tumor genetic profile data, which is increasingly central to tumor prognostication [3]. Likewise, the application of standard MRI is limited for response assessment and treatment monitoring, because it cannot distinguish between treatment effects (including radiation necrosis) and tumor progression with high accuracy. Given these limitations, there is clearly a need for more sophisticated advanced neuroimaging modalities that can augment and resolve the gaps in the existing standard imaging. The advanced noninvasive neuroimaging modalities whose clinical use is emerging in response to this need include amino acid positron emission tomography (aaPET) and advanced MRI (aMRI). Especially as hybrid PET/MRI imaging systems become available in more clinical settings [4], the opportunities to use these two important modalities within the same exam are likely to facilitate profound advances in neurooncologic imaging capabilities.

PET is a noninvasive imaging modality that involves administration of a radioactive (positron-emitting) tracer and subsequent photon detection by a PET scanner to quantify radioactivity accumulation in tissue. Depending on which tissue accumulates the tracer or its metabolites, the PET scan will reveal a differential profile of radioactivity. aaPET tracers provide information pertaining to the tissue, depending on the transport and metabolism of that specific amino acid molecule. aaPET imaging has been endorsed by the Response Assessment in Neuro-Oncology Working Group and the European Association for Neuro-Oncology for diagnosing and grading tumors, delineating extent for resection or radiation planning, monitoring treatment, and diagnosing progression in gliomas and certain metastatic brain tumors [5–7]. The main radiotracers for aaPET in neurooncology include L-[methyl]-^11^C-methionine (MET), ^18^F-fluoroethyl-tyrosine (FET), ^18^F-fluoro-L-dihydroxy-phenylalanine (FDOPA), and ^11^C-alpha-methyl-L-tryptophan (AMT). MET was the first aaPET radiotracer developed and widely used [8]; however, the 20 min half-life of the carbon-11 isotope limits its widespread clinical utility. FET was developed based on tyrosine transport from blood to tumor tissues, and the use of fluorine-18 with 110 min half-life in this tracer makes FET much more amenable for routine clinical use and distribution [9, 10]. FET has been widely adopted and is approved for clinical neurooncology use in several European countries. FDOPA was a tracer originally developed for imaging the basal ganglia [11, 12], as it measures dopamine synthesis, but it has recently been adopted for neurooncologic imaging, as it utilizes the same L-type amino acid transporter system as other aaPET tracers [13]. AMT was originally developed for imaging serotonin synthesis [14], and it was later adopted for imaging epileptic tissue [15] and CNS neoplasms [16, 17]. A unique aspect of AMT PET is its potential to image the activity of the immunosuppressive kynurenine pathway [13, 18]. There are additional aaPET radiotracers under development and investigation, but we will limit our scope in the present review to these four main tracers while focusing on the most recent developments.

The leading aMRI techniques for clinical neurooncology imaging are diffusion-weighted imaging (DWI), perfusion-weighted imaging (PWI), and magnetic resonance spectroscopy (MRS). DWI creates tissue contrast reflective of the random, microscopic translational motion of water molecules in the body. The diffusivity is measured by apparent diffusion coefficient (ADC). In tumors, free diffusivity of water molecules can be impaired by high cell density and/or high nuclear-to-cytoplasmic ratio; thus, reduced diffusivity is a surrogate marker for increased tumor cellularity [19]. DWI can improve detection, characterize the biology of tumors, and monitor treatment response; it is also being investigated for tumor grading [20]. PWI is used to noninvasively measure cerebral perfusion, through hemodynamic measurements like cerebral blood volume, blood flow, and mean transit time [21]. The PWI techniques of dynamic susceptibility contrast (DSC) and arterial spin labeling (ASL) operate on similar physical principles, with tagged blood causing a transient, localized change of the MR relaxation rates depending on perfusion properties. However, DSC uses an exogenous contrast material and ASL uses arterial water as an endogenous tracer. PWI has been mainly used in stroke assessment but also found a useful clinical application for identifying and grading brain tumors [22], such as with cerebral blood volume maps to assess the neovascularity of tumors, which may correlate with tumor grade and malignant histology [21]. MRS is used to create a nuclear magnetic resonance spectrum of the chemical content of tissue, which allows for noninvasive assessment of the molecular composition of tumors [23]. MRS may thus be used alongside standard MRI to improve brain tumor diagnostic accuracy and assessment of treatment changes [23]. With MRS, there is a specific molecular signature (the spectrum) related to both the interaction of the magnetic field with each molecule's unique distribution of electrons and the interactions between nuclei. The areas of peaks within the spectrum are associated with (but not a direct measure of) concentration. This allows for noninvasive assessment of molecular composition of tumors, including several important metabolites. As tumor grade increases, the metabolites N-acetylaspartate and creatine decrease, while the metabolites choline, lipids, and lactate increase, resulting in changes to the ratios between peak areas [24].

As these two distinct advanced noninvasive neuroimaging modalities have evolved in recent years, there has been a growing interest in better characterizing the unique strengths of each modality, how aMRI and aaPET techniques compare, and how they might be used in a complementary manner to elucidate a more complete understanding of a brain tumor's profile. The recent review article by Lohmann et al. [25], focusing on studies completed prior to 2018, established a basis for understanding these imaging modalities in a neurooncologic context. In four studies that combined MRS with aaPET, one with MET PET [26] and three with FET PET [27–29], a high diagnostic accuracy of the individual and combined modalities was reported, but with inconsistent spatial congruence [25]. In 13 comparative studies of PWI with aaPET, seven used FET PET with DSC PWI [30–36], four used MET PET with DSC PWI [26, 37–39], one used FDOPA PET with DSC PWI [40], and one used FDOPA PET with ASL [41]. Overall, aaPET was superior to PWI for diagnosing recurrent glioma, and PWI did not accurately represent the extent of glioma, although there was some variability between studies using different methodology about whether the modalities resulted in spatially congruent images. The seven studies of DWI combined with aaPET, two that used FDOPA PET [42, 43], three with MET PET [44–46], and two that used FET PET [36, 47], showed mixed results. Overall, there were contradictory results as to whether aaPET uptake was congruent with DWI diffusion coefficient, but aaPET was overall superior for estimating tumor cell density and for differentiating tumor progression from treatment-related changes. Lohmann and colleagues [25] noted that aaPET benefits from robust interinstitution reliability, whereas aMRI is subject to considerable standardization issues. They concluded that the conflicting results of studies reviewed indicate that aaPET and aMRI encode different biological properties, and further research is needed, especially with correlates to neuropathology, to understand how these modalities can be collectively leveraged. Notably, their review did not include the fourth major aaPET tracer, AMT, which has been tested for various neurooncology applications over the past decade.

Overall, there is significant variability in the published results of aaPET and aMRI comparisons, and further investigation of these combined modalities is warranted. In the present review, we summarize the research progress for aaPET and aMRI in for neurooncology imaging, while focusing on reports published in the past 2 years. Specifically, this systematic review considers only those studies that directly compare and/or combine these modalities (aaPET and aMRI). We include the most recent data on the main aaPET tracers and aMRI sequences, as well as some emerging MRI sequences not included in previous review articles.

## 2. Methods

**Figure 1:**
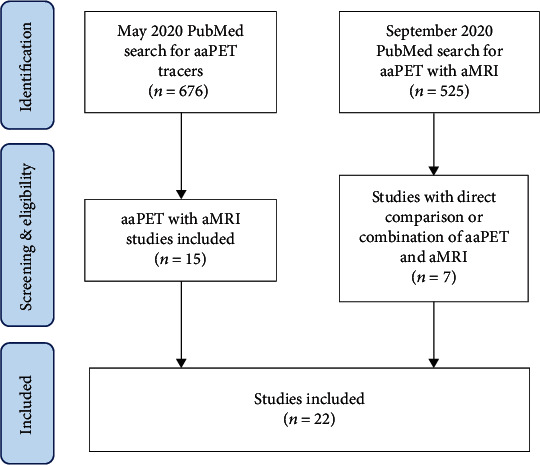
PRISMA study selection flowchart.

This review included original clinical research studies of either direct comparison of aaPET versus aMRI or combination studies of aaPET with aMRI, in neurooncology applications, published between July 2018 and August 2020. The PubMed database was searched through two mechanisms to identify such publications (Figure 1). This study timeframe was chosen with several months overlap to the previous review, in acknowledgement of the delay between publication and PubMed indexing.

The first search was completed in the end of May 2020, covering the January 2014–May 2020 timeframe, using search terms “amino acid PET brain.” A total of 676 records were initially identified using these search terms. These records were then screened for original research publications related to direct comparison of aaPET versus aMRI, or combination study of aaPET with aMRI, in neurooncology, including retrospective and prospective clinical studies, but excluding case reports, review papers, systematic reviews, and meta-analyses. This yielded 15 studies published during the period of July 2018–May 2020.

The second search was completed in September 2020, covering the July 2018–August 2020 timeframe, using the search terms adopted from Lohmann et al. [25]: ((glioblastoma) OR (brain tumors) OR (high-grade glioma)) AND ((positron emission tomography) OR (PET) OR (amino acid PET)) AND ((magnetic resonance imaging) OR (magnetic resonance spectroscopy) OR (perfusion-weighted imaging) OR (diffusion-weighted imaging) OR (chemical exchange saturation transfer) OR (MRI) OR (advanced MRI) OR (MRS) OR (PWI) OR (DWI) OR (CEST) OR (hybrid PET/MR)). This yielded a total of 525 records, which were then screened for original research publications related to direct comparison of aaPET versus aMRI, or combination study of aaPET with aMRI, in neurooncology, including retrospective and prospective clinical studies, but excluding case reports, review papers, systematic reviews, meta-analyses, and papers identified through the first search process. This yielded additional 7 studies, for a total of 22 studies, which were included in the final review (see study selection flowchart in Figure 1 and key study details summarized in Tables 1–4). Data collection and extraction was performed independently by one author (BS) with oversight by the other authors. Data variables collected include year, study location, number of patients, aaPET tracer(s) studied, aMRI technique(s) studied, tumor type, and main study results. Data was analyzed and summarized qualitatively. Of note, combined PET and MRI scans not acquired simultaneously on a hybrid PET/MRI are deemed to have been performed asynchronously.

## 3. Results

### 3.1. Amino Acid PET and Diffusion-Weighted MR Imaging (Table 1)

#### 3.1.1. MET PET with DWI

MET PET was compared with DWI in a recent study of 124 glioma patients [48]. Scans were performed asynchronously before surgery for 47 high-grade and 77 low-grade gliomas and compared with reference standard histopathology for diagnostic accuracy. DWI had a sensitivity 80.9%, specificity 59.7%, and accuracy (in terms of AUC) 70.3% for differentiating high- from low-grade glioma, and MET PET had sensitivity 95.7%, specificity 41.6%, and accuracy of 68.7%. With combined modalities, the authors found that, for DWI-negative gliomas, MET PET demonstrated higher uptake in IDH-1/2wt gliomas. Moreover, both imaging modalities were significant predictors of progression-free survival. Overall, MET PET was found to be more sensitive than DWI for detecting high-grade glioma by visual analysis, though these modalities reached comparable accuracy. Notably, in the patients with negative DWI, the role of MET PET becomes more relevant, perhaps due to the shift in tumor cell density associated with altered water distribution. Although it did not include ADC maps, this multimodality characterization of gliomas builds upon the previous literature, which found MET PET to be more robust than DWI [25], probably reflecting the fact that the modalities encode different biological properties.

#### 3.1.2. FET PET with DWI

**Figure 2:**
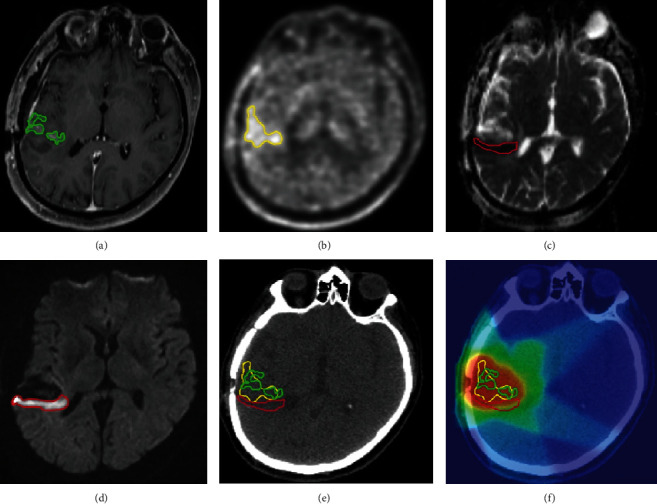
FET PET combined with DWI in recurrent glioblastoma (histologically confirmed). (a) Axial postcontrast T1-weighted MRI, green: gross total volume (GTV). (b) Axial FET PET, yellow: GTV on PET. (c) ADC map, red: GTV by ADC (low), corresponding to a hyperintensity in (d) DWI-MRI. (e) Illustration of all three volumes on planning CT. (f) Dose distribution of the treatment plan based on FET PET. The volumetric comparison thus revealed that the majority of the GTVs-ADC (low) is located outside areas of contrast enhancement in postcontrast T1-weighted MRI (mean nonoverlapping volume 66.2 ± 24.6%, median 72.2%, and range 5.8–100%) and also outside areas of increased FET uptake (mean nonoverlapping volume 76.4 ± 64%, median 69.5%, and range 12.3–385.1% in the total cohort, *n* = 41). Reproduced with permission from Figure 3 in Popp et al. [47].

FET PET and DWI modalities have been studied jointly in glioma and glioblastoma (GBM) patients in several recent direct comparison reports. In one study of 41 recurrent GBMs, Popp et al. compared tumor volume and localization between contrast-enhanced T1-weighted MRI, FET PET, and DWI, scanned asynchronously [47]. The tumor volumes derived from postcontrast T1-weighted MRI and FET PET were better overlapped with recurrence after reirradiation than those from DWI. The tumor volume from FET PET was larger than that from T1-weighted MRI, which was larger than that of DWI (*P* < 0.001) (Figure 2).

DWI also demonstrated more nonoverlapping area than overlapping area, compared with T1-weighted MRI and FET PET (*P* < 0.001), such that including DWI volume would add up to 48.5% to the tumor volume [47]. This highlights the complementary nature of these imaging modalities, which capture distinct biological tumor properties, although this study did not correlate the imaging findings with histopathology tissue analysis.

Another small study of 16 GBMs compared FET PET and DWI performed on the same day, for predicting tumor recurrence [49]. FET uptake was the only parameter that could differentiate recurrent tumor volume across all tissue types (white matter, gray matter, contrast-enhancing, and nonenhancing lesions). Other parameters demonstrated tumor discrimination, but their effects were tissue-dependent. Combining all PET (FET and ^18^F-fluoro-deoxy-glucose [FDG]) and MRI (DWI and PWI) parameters together yielded AUC 0.73 in white matter, 0.78 in gray matter, 0.68 in contrast-enhancing lesions, 0.72 in nonenhancing lesions, and 0.77 overall. They concluded that combining parameters could provide patient-specific maps of probability of recurrence, and FET PET had the highest overall predictive value.

**Figure 3:**
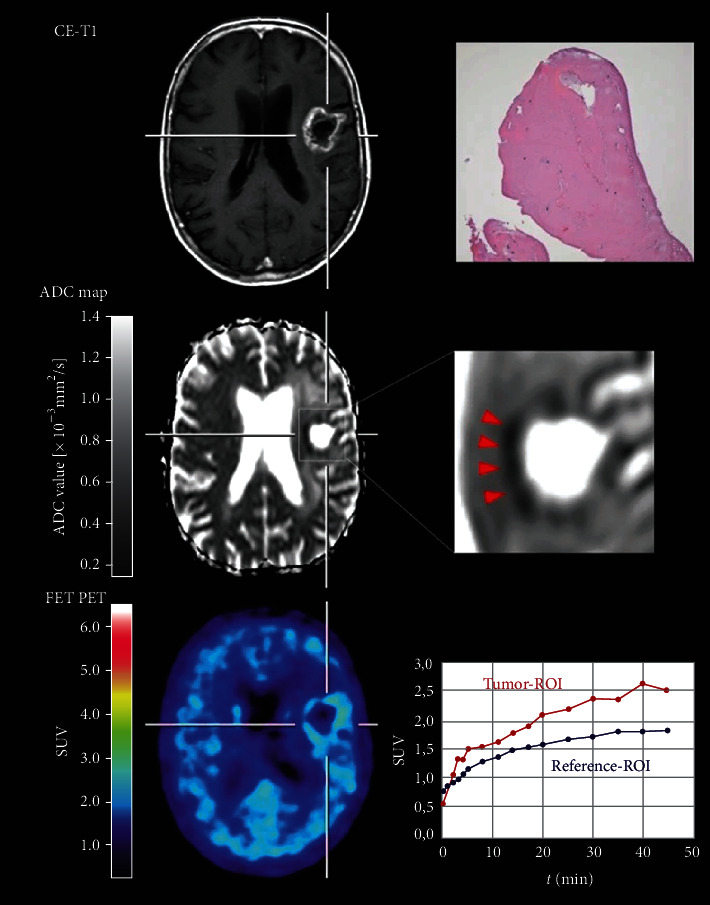
FET PET combined with DWI to detect radiation necrosis (histologically confirmed) after glioma treatment. Contrast-enhanced MRI, ADC map obtained from DWI, and FET PET of a 69-year-old female patient with anaplastic oligodendroglioma. Twenty-nine months after fractionated external beam radiotherapy, brachytherapy, and adjuvant temozolomide chemotherapy, contrast-enhanced MRI suggested tumor progression. In spatial correspondence to the contrast enhancement, the ADC map revealed a substantial decrease of diffusivity in the area of contrast enhancement (arrowheads on the enlarged image; ADC below 0.5 × 10^−3^ mm^2^/s), suggesting tumor progression. In contrast, FET PET showed no increased metabolic activity and a steadily increasing time-activity curve, indicating treatment-related changes. Histological findings obtained following stereotactic biopsy were consistent with radiation necrosis (hematoxylin and eosin staining: original magnification, ×200; scale bar, 50 *μ*m). For a follow-up time of 6 years, the patient was in a stable clinical condition. Reproduced with permission from Figure 1 of Werner et al. [50].

In a study of 48 high-grade gliomas with suspected progression, Werner et al. compared FET PET with DWI for differentiation of treatment-related changes from tumor progression [50]. DWI and FET PET were obtained simultaneously in 11 patients and asynchronously in 37 patients, and diagnosis was confirmed either by neuropathology (79%) or clinicoradiologically (21%). FET PET performed better (accuracy 83%) than DWI (accuracy 69%) (Figure 3), and when combined, static FET PET plus DWI had an accuracy of 89%, while dynamic FET PET plus DWI had the highest accuracy of 93%.They also observed that FET PET parameters were significant predictors of survival time, whereas DWI was not. Overall, the authors concluded that FET PET should be preferred over DWI for differentiating tumor progression form treatment-induced tissue changes [50].

Similarly, Lohmeier et al. studied the comparative ability of FET PET and DWI to differentiate between recurrent glioma and treatment-related effects in 42 glioma patients [51]. Using simultaneous PET/MRI acquisition, they found that both modalities have reliable diagnostic performance: FET PET (AUC 0.81, sensitivity 81%, and specificity 60%) and DWI (AUC 0.82, sensitivity 62%, and specificity 100%). Their combined performance in a biparametric approach had the highest diagnostic accuracy (AUC 0.90, sensitivity 97%, and specificity 60%), though there was no statistically significant difference in diagnostic power. The benefit of combining modalities was especially important when the FET PET parameter of tumor-to-brain ratio maximum was close to threshold, in which case the addition of the DWI parameter ADC-mean effectively improved clinical detection.

In Verburg et al., 20 newly diagnosed gliomas were scanned with FET PET and DWI asynchronously before treatment [52]. They compared the ability of each imaging modality to detect tumor as compared to neuropathology confirmation. For nonenhancing gliomas, DWI with T1-weighted MRI yielded the best tumor detection (AUC 0.90) and had the highest prediction accuracy (88%). FET PET was not included in the optimal combination for nonenhancing glioma and in fact had lower diagnostic accuracy than standard fluid-attenuated inversion recovery (FLAIR) MRI. Further subtype analysis found that the highest accuracy for diagnosing high-grade IDH-wild type (IDH-wt) FET-positive glioma was using DWI combined with FET PET (AUC 0.89), and for FET-negative gliomas, the DWI parameters had the highest diagnostic accuracy (AUC 1.00). They conclude that enhancing glioma infiltration is best detected by a combination of DWI with FET PET, and importantly, although FET PET is part of the optimal imaging combination for most brain tumors, it is not for nonenhancing gliomas. This finding is unique among FET PET with DWI studies, which largely support FET PET as the superior imaging modality, because with nonenhancing gliomas, DWI performs better.

#### 3.1.3. FDOPA PET with DWI

In a study of treatment-naïve gliomas, Tatekawa et al. imaged 63 patients with DWI and FDOPA PET, within 2 months of each other [53]. They found a negative correlation between FDOPA PET SUV and DWI ADC in IDH-wt gliomas, both on voxel-wise (*r* = −0.19) and patient-wise (*r* = −0.58) analysis. There was a negative correlation in IDH-mutant-1p/19q-non-codeleted (IDH_m-non-codel_) gliomas, both on voxel-wise (*r* = −0.19) and patient-wise (*r* = −0.61) analysis. In IDH-mutant-codeleted (IDH_m-codel_) gliomas, there was a positive correlation only on voxel-wise analysis (*r* = 0.18). The *r* value on voxel-wise analysis was significantly higher in IDH_m-codel_ than in IDH-wt or IDH_m-non-codel_ (*P* < 0.001), but it was not significantly different between molecular groups on patient-wise analysis. On receiver operating curve (ROC) analysis, IDH_m-codel_ was differentiated from IDH-wt and IDH_m-non-codel_ with an AUC of 0.80 (sensitivity 63% and specificity 92%). On Cox multivariate analysis of IDH-wt glioma, there was a significant voxel-wise association (HR = 0.085, *P* = 0.038). And on the log-rank tests of IDH-wt gliomas, there was a significant difference in overall survival when IDH-wt glioma was stratified by the voxel-wise *r* value, with lower *r* value being associated with worse survival [53].

Piccardo et al. scanned 22 pediatric diffuse midline gliomas (DMGs) with an asynchronous multimodal imaging approach [54]. They demonstrated a strong correlation between FDOPA tumor-to-striatum uptake ratio and DWI relative minimum ADC (*P* < 0.01). Both parameters were able to successfully differentiate low- and high-grade DMGs (*P* < 0.01): FDOPA PET tumor-to-striatum ratio provided AUC 0.94 (sensitivity 83% and specificity 60%), FDOPA PET tumor-to-brain ratio gave AUC 0.82 (sensitivity 91% and specificity 80%), while DWI relative minimum ADC yielded AUC 0.81 (sensitivity 83% and specificity 70%). However, the only parameter to significantly differentiate between H3K27M-mutant and wild-type DMGs independent of histology was FDOPA uptake ratio (*P* = 0.003). DWI did not reach significance for differentiating H3K27M-mutant status (*P* = 0.21) [54].

#### 3.1.4. AMT PET with DWI

**Figure 4:**
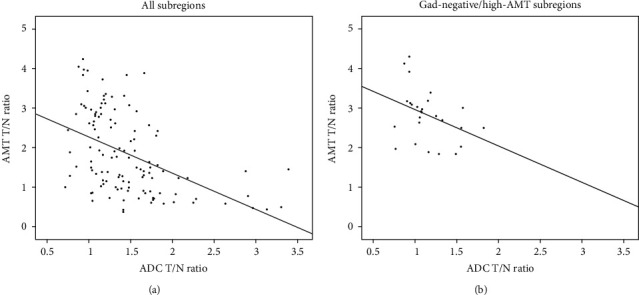
Correlation between AMT uptake and ADC values in glioblastoma subregions. (a) Negative correlation in the whole set of tumor subregions (*N* = 115, including tumor regions with both high and low AMT uptakes; Spearman's rho [*r*] = −0.52, *P* < 0.0001). (b) A similar trend was detected in noncontrast-enhancing/high-AMT tumor subregions, consistent with tumor-infiltrated brain (*N* = 25, *r* = −0.40, *P* = 0.05). Reproduced with permission from Figure 3 of John et al. [17].

A comparative study of AMT PET with DWI, by John et al., scanned 30 newly diagnosed GBMs, with an average 3 days between DWI MRI and AMT PET scans [17]. They identified a strong negative correlation between AMT standardized uptake values (SUVs) and DWI-derived ADC (*P* < 0.0001) (Figure 4). Areas beyond contrast enhancement on MRI that demonstrated high AMT PET uptake were associated with low ADC on DWI (*P* = 0.05), and these areas were indicative of tumor-infiltrated brain. In nonenhancing T2w/FLAIR hyperintense areas, there was also a correlation between low AMT PET uptake with high ADC values, which indicates peritumoral vasogenic edema. Only AMT PET uptake was associated with overall survival (HR 7.8, *P* = 0.003), and high uptake ratio was also predictive of the location of posttreatment tumor progression [17].

This study built on a previous study of the same group that combined AMT PET with DWI-derived isotropic diffusion spectrum imaging, which detected metabolically active glioma regions with high cellularity and differentiated high- vs. low-grade gliomas accurately albeit in a small cohort (*n* = 10) [55].

### 3.2. Amino Acid PET and Perfusion-Weighted MR Imaging (Table 2)

#### 3.2.1. MET PET with PWI

MET PET imaging was compared to PWI MRI in several studies over the past two years. Beppu et al. scanned 24 recurrent GBMs asynchronously using MET PET and ASL perfusion imaging before and 4 and 8 weeks after treatment initiation [56]. MET PET tumor volume was significantly larger than that of ASL at baseline, but no significant difference was found after treatment. MET PET and ASL parameters were significantly correlated at all time points. Each modality demonstrated moderate predictive power for progression-free survival: MET PET had AUC 0.44 at baseline, 0.66 at 4 weeks, and 0.73 at 8 weeks; ASL had AUC 0.41 at baseline, 0.65 at 4 weeks, and 0.66 at 8 weeks. ASL could predict progression-free survival, but the most accurate predictor was MET PET, with 76.9% sensitivity and 81.8% specificity at 8 weeks. The authors concluded that these modalities provide different information; however, they did not study the performance of these imaging modalities combined [56]. In another study of 18 GBMs, Pala et al. obtained MET PET and PWI scans asynchronously before, during (MRI only), and after surgery in order to compare tumor volume and diagnostic accuracy compared to histopathology [57]. Similar to Beppu et al., they found that before gross total resection, MET PET yielded a larger tumor volume than PWI, a difference that disappeared after surgery. MET PET demonstrated higher sensitivity for GBM detection (95%) than PWI (67%). The authors did not explore the question of combining modalities [57].

**Figure 5:**
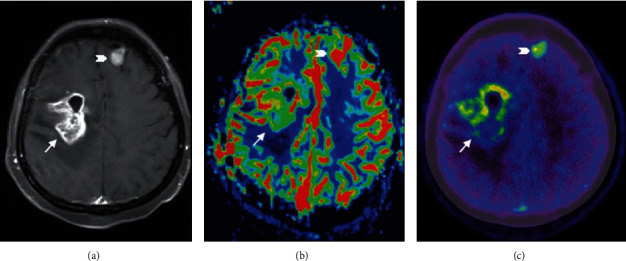
MET PET combined with PWI in recurrent glioblastoma. Contrast-enhanced T1-weighted MR image (a) and a relative cerebral blood volume (CBV) map (b) and a MET PET/CT image (c) for a 41-year-old woman with recurrent glioblastoma. The lesion in the right frontal lobe (arrow) shows enhancement (a) and positive findings on both the rCBV map (b) and MET PET/CT image (c). The lesion localized in the left frontal lobe (chevron) might be a meningioma, which has been stable for several years. Reproduced with permission from Figure 2 of Qiao et al. [58].

**Figure 6:**
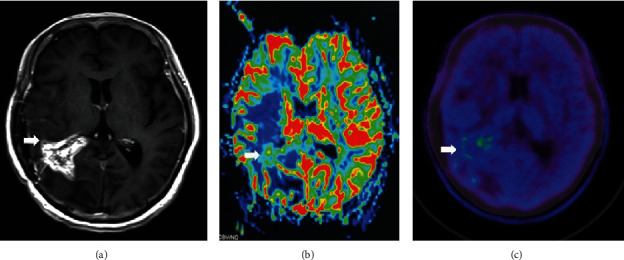
MET PET with PWI of radiation necrosis. Contrast-enhanced T1-weighted MR image (a) and relative CBV map (b) and MET PET/CT image (c) for a 44-year-old man with necrosis. The lesion in the right parietal lobe (arrow) shows enhancement (a) and negative findings on both the rCBV map (b) and the MET PET/CT image (c). Reproduced with permission from Figure 3 of Qiao et al. [58].

Comparing MET PET to DSC PWI for their ability to differentiate radiation injury from recurrent tumor, Qiao et al. scanned 42 high-grade gliomas asynchronously [58]. All parameters were shown to differ significantly between recurrent tumor tissue and radiation injury tissue. MET PET yielded AUC 0.847, sensitivity 90.9%, and specificity 55.6%, while PWI yielded AUC 0.845, sensitivity 66.7%, and specificity 77.8%. Combining imaging modality parameters resulted in the largest AUC, 0.953, with sensitivity 84.8% and specificity 100% (Figures 5 and 6). They concluded that the combination of these modalities yielded the best diagnostic accuracy to differentiate recurrence from radiation injury in high-grade glioma [58].

Studying these imaging modalities in oligodendrogliomas, Roodakker et al. compared MET PET and PWI images acquired asynchronously, with histology as the ground truth, in four oligodendrogliomas [39]. MET PET uptake was significantly correlated to tumor cell density, but PWI was not correlated with MET PET or histology. They concluded that MET PET, but not PWI, has a value as an indicator of tumor cell density in oligodendrogliomas. While in a very small study, this result differs from that of the GBM studies above and may indicate an important tumor type difference, wherein the value of PWI may be negligible for oligodendrogliomas specifically [39].

#### 3.2.2. FET PET with PWI

Advances have also been made in comparing FET PET to PWI in the past two years. Lundemann et al. compared FET PET and PWI for predicting recurrence in 16 GBMs [49]. FET uptake was the only parameter that could significantly differentiate recurrence in all tissue types, while PWI parameters had a tissue-dependent effect, in white matter, gray matter, and nonenhancing lesion. When combined, FET PET, DWI, PWI, and FDG PET/MRI parameters together achieved AUC 0.73 in white matter, 0.78 in gray matter, 0.68 in contrast-enhancing lesions, 0.72 in nonenhancing lesions, and 0.77 overall. They concluded that FET PET had the strongest predictive value for recurrence in GBM [49].

FET PET and PWI have been investigated in high-grade gliomas as well. Dissaux et al. assessed these modalities asynchronously in 30 patients, to assess tumor volume delineation compared to standard MRI [59]. Using scans obtained an average of 6 days apart, they found that FET PET tumor volume was significantly larger than volume on standard MRI (*P* = 0.005) with a low overlap volume, whereas PWI volume was significantly smaller than standard MRI volume (*P* < 0.001) with a high overlap volume. They concluded that PWI parameters were highly correlated with standard MRI, whereas FET PET provided complementary information. This analysis is limited by the lack of histologic confirmation and the lack of direct comparison or combination analysis of FET PET with PWI [59].

Verburg et al., in their multimodal imaging analysis of 20 newly diagnosed gliomas, compared FET PET with PWI acquired asynchronously before treatment, to compare diagnostic accuracy [52]. In IDH-mutant gliomas, FET PET combined with DSC PWI yielded an AUC 0.82. They did not report on this imaging combination for any other tumor subtypes. In another study of 46 newly diagnosed gliomas, Schon et al. [60] found the tumor volume to be larger by FET PET than by DSC PWI. For identifying cellularity, FET PET parameters performed the strongest, while for vascularity, both DSC PWI and FET PET performed well. The authors noted that in FLAIR hyperintense GBM regions, PWI parameters were significantly higher in the FET PET-positive areas (*P* < 0.001) [60].

Similar to the previous literature, which found variability in spatial correlation of aaPET with PWI, these new data also show inconsistent results. In high-grade gliomas, there was little overlapping volume between these imaging modalities, but in newly diagnosed gliomas, the high FET PET uptake regions had increased PWI parameters. There may be tumor type differences or PWI sequence differences contributing to these discrepancies.

#### 3.2.3. FDOPA PET with PWI

In a study of 40 gliomas, Fraioli et al. scanned patients with FDOPA PET and DSC PWI synchronously, with tumor confirmed on biopsy, to assess the ability of these modalities to identify tumor features [61]. PWI parameters correlated with tumor grade (*P* < 0.001) but not with FDOPA PET parameters. FDOPA PET maximum SUV analysis was able to distinguish enhancing and nonenhancing tumor components from necrosis and normal tissue. FDOPA PET and PWI each achieved an AUC 0.94 for differentiating tumor tissue, and when combined, they achieved a high AUC of 0.99.

In a study of treatment-naïve gliomas, Tatekawa et al. imaged 61 patients with PWI and FDOPA PET, within 2 months of each other [53]. They found in IDH-wt gliomas a positive correlation between FDOPA PET SUV and PWI relative cerebral blood flow (rCBV), both on voxel-wise (*r* = 0.25) and patient-wise (*r* = 0.50) analysis. There was a positive correlation in IDH_m-non-codel_ gliomas, both on voxel-wise (*r* = 0.31) and patient-wise (*r* = 0.70) analysis. There was no correlation in IDH_m-codel_ gliomas. The *r* value on voxel-wise analysis was significantly lower in IDH_m-codel_ than in IDH-wt or IDH_m-non-codel_ (*P* < 0.01), but it was not significantly different between molecular groups on patient-wise analysis. On ROC analysis, IDH_m-codel_ was differentiated from IDH-wt and IDH_m-non-codel_ with an AUC of 0.68 (sensitivity 69% and specificity 73%). Weak voxel-wise correlation between FDOPA SUV and rCBV or ADC had a significant association with better overall survival in IDHwt gliomas. The authors explained this by a mechanism where gliomas with heterogeneous features show weak correlations between physiological MRI/PET, have less efficient growth, and have better prognosis.

#### 3.2.4. AMT PET with PWI

**Figure 7:**
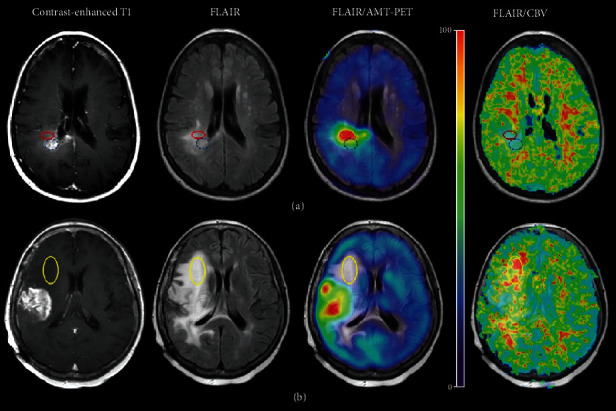
Spatial mismatch between AMT PET uptake and relative cerebral blood volume (CBV) on PWI. Examples of ^11^C-alpha-methyl-L-tryptophan (AMT) and relative cerebral blood volume (rCBV) mismatch in patients with newly diagnosed (a) and recurrent (b) glioblastoma. (a) In patient #6, high AMT uptake (AMT tumor to normal [T/N] ratio: 2.78) was associated with low rCBV values (T/N ratio: 0.88) (red circles) adjacent to the contrast-enhancing tumor mass (blue dashed circles). (b) In patient #15, low AMT uptake (T/N ratio: 0.54) was associated with increased rCBV values (T/N ratio: 1.60) in the extensive fluid-attenuated inversion recovery (FLAIR) hyperintense area surrounding the contrast-enhancing tumor mass with high-AMT uptake (yellow circle). The color bar shows a relative scale (0%-100%). Reproduced with permission from Figure 3 of John et al. [62].

In the first comparative study of AMT PET with PWI, John et al. scanned 20 GBMs asynchronously with an average of 3 days between scans to evaluate characteristics of the nonenhancing tumor regions [62]. AMT PET and PWI demonstrated a moderate positive correlation in T2w/FLAIR hyperintense regions (*r* = 0.41 and *P* = 0.017), which was stronger for recurrent tumors (*r* = 0.55 and *P* = 0.034) than for newly diagnosed GBM (*r* = 0.23 and *P* = 0.37). This demonstrates an overall moderate correlation between AMT PET and PWI in nonenhancing GBM regions, and a spatial mismatch between AMT uptake and relative cerebral blood volume was often present (Figure 7).

Tumor regions with very low rCBV (below 0.79 tumor/normal ratios), measured by PWI, showed invariably low AMT uptake. AMT PET was able to detect metabolically active tumor portions in nonenhancing (T2w/FLAIR hyperintense) tumor regions if the rCBV values were above the 0.79 threshold [62].

### 3.3. Amino Acid PET and MR Spectroscopy (Table 3)

#### 3.3.1. MET PET with MRS

Two recent studies compared MET PET with MRS. Kudulaiti et al. retrospectively reviewed the scans of 109 nonenhancing supratentorial lesions with MET PET and MRS prior to treatment, and compared their ability to differentiate glioma from nonglioma lesions [63]. The Cho/NAA index (CNI) for each voxel was used as the MRS parameter. Pathology revealed that the most common tumor types were astrocytoma (38.5%), oligodendroglioma (16.5%), and anaplastic astrocytoma (16.5%). MRS reached a sensitivity of 60% and specificity of 50%, whereas MET PET reached a higher sensitivity of 75.8% and a specificity of 50%, a statistically significant difference. With both PET and MRS combined, the sensitivity rose to 89.5%, which was significantly higher, and specificity dropped to 42.9%, which was not significant.

Kebir et al. scanned 19 histologically confirmed gliomas with MET PET and MRS in a single standardized imaging session, prior to treatment, and compared their ability to classify glioma molecular subtypes [64]. The spectroscopy peaks for NAA, choline, and Cr were used as the MRS parameters. MET PET uptake was superior for identifying IDH status (AUC 0.67), with a tumor-to-brain uptake ratio higher in IDH-wt gliomas (3.61) than in IDH-mutant gliomas (2.37). MRS was superior for glioma subgrouping of IDH-wt GBM, IDH-wt grade II/III glioma, and IDH-mutant grade II/III glioma with and without 1p/19q codeletion (AUC 0.68). These modalities performed better individually, as the combined modalities yielded AUC 0.61 for classifying glioma subtypes and 0.58 for classifying IDH status. Together, these studies show that MET PET and MRS individually and together have diagnostic potential for diagnosing glioma, and differentiating high-grade from low-grade, but that they perform relatively poorly for classifying glioma molecular subtype. This extends upon the previous literature, which reported that both MET PET and MRS hold high diagnostic accuracy for diagnosing high-grade glioma recurrence.

#### 3.3.2. FET PET with MRS

Recent comparisons of MRS data with FET PET have been less fruitful. There were two studies that combined FET PET with MRS; however, one study attempted 3D MRS imaging with a quality insufficient for analysis [49], while the other study found that imaging measurements of MRS were missing from a large number of biopsy samples as the voxels did not include brain regions where the biopsy was originated from [52]. As such, there are no new successful advances in FET PET with MRS, contrary to the review by Lohmann et al. [25], which found several reports supporting the conclusion that FET uptake was significantly correlated with MRS metrics, although they were spatially incongruent and represented complementary information. It may be that because both recent studies were taking a multimodal imaging analysis approach, the relatively weak or incomplete data from MRS were excluded in favor of focusing on the other stronger modalities. Future research efforts focused specifically on the utility of whole-brain MRS imaging [65], providing more widespread sampling ability, with aaPET may be able to overcome such limitations.

#### 3.3.3. FDOPA PET with MRS

**Figure 8:**
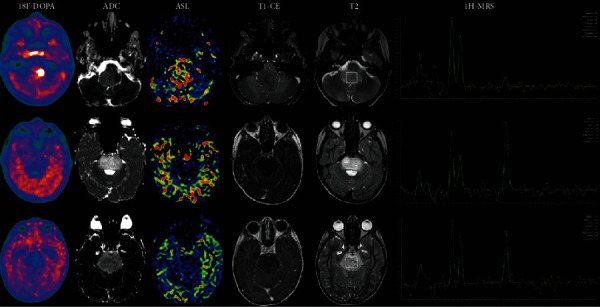
FDOPA PET combined with MRS in diffuse midline glioma (DMG). ^18^F-DOPA PET and MR images of H3K27M-mutant and wild-type DMG. Upper row: DMG, H3K27M-mutant, and WHO grade IV; ^18^F-DOPA PET shows an area of markedly increased uptake within the lesion (tumor/normal [T/N] ratio: 2.80; tumor/striatum [T/S] ratio: 1.80). ADC demonstrates a focal area with mildly reduced diffusivity (rADC min: 0.95) on the left side of the lesion corresponding to the ^18^F-DOPA PET hot spot region. ASL shows increased perfusion (rCBF max: 1.90). Contrast-enhanced (CE) T1-weighted MR image does not show contrast enhancement. ^1^H-MRS demonstrates marked increase of Cho/NAA (7.15) and mild increase of Cho/Cr (1.39) ratios. Middle row: diffuse intrinsic pontine glioma, H3K27M-wild type, and WHO grade III; ^18^F-DOPA PET shows absence of tracer uptake in the lesion (T/N: 1.00; T/S: 0.60). ADC demonstrates increased diffusion (rADC min: 1.42). ASL shows low perfusion (rCBF max: 0.80). CE T1-weighted MR image does not show contrast enhancement. ^1^H-MRS demonstrates normal Cho/NAA (0.80) and mild increase of Cho/Cr (1.54) ratios. Of note, this was the only histologically defined high-grade glioma which demonstrated lack of increased ^18^F-DOPA uptake. Lower row: Diffuse intrinsic pontine glioma, H3K27M-wild type, and WHO grade II. ^18^F-DOPA PET shows absence of tracer uptake in the lesion (T/N: 0.95; T/S: 0.45). ADC and ASL images demonstrate increased diffusion (rADC min: 1.26) and low perfusion (rCBF max: 0.78). CE T1-weighted MR image does not show contrast enhancement. ^1^H-MRS demonstrates normal Cho/NAA (0.93) and Cho/Cr (1.08) peak area ratios. Note: the box on T2-weighted images indicates the region of interest from which the spectra were acquired. Reproduced with permission from Figure 1 of Piccardo et al. [54].

FDOPA PET has also been studied alongside MRS in a recent retrospective review of 22 pediatric DMGs [54]. In this multimodal imaging study, the authors compared, among others, FDOPA PET to MRS using scans obtained two weeks apart and compared the diagnostic potential of each modality to histology and molecular analysis. The MRS parameters used were choline-to-creatine peak area ratio, choline-to-N-acetylaspartate peak area ratio, and lactate peak. FDOPA PET and MRS parameters were both able to differentiate low-grade from high-grade DMG and to differentiate between H3K27M-mutant and wild-type DMG (Figure 8).

FDOPA PET outperformed MRS on ROC analysis, with AUC 0.94 compared to 0.78, respectively. FDOPA uptake was the only parameter that could discriminate H3K27M-mutant from wild-type DMG independently of histology (AUC 0.91). Overall, FDOPA PET was more diagnostic for pediatric DMG, although they did not attempt to combine modalities for improved diagnostic ability. Future research efforts may thus be targeted towards comparing these modalities in different tumor types and exploring the possibility of improved diagnostics with combined imaging parameters.

### 3.4. Emerging aMRI Techniques Combined with aaPET (Table 4)

There are several aMRI imaging techniques that are emerging in response to the need for improved neuroimaging. These techniques are not well-developed or researched enough to include in the above discussions, but we include them here as they may prove to be valuable as more data are published. We summarize here those emerging aMRI techniques that are sufficiently developed such that a comparative aMRI with aaPET study in neurooncology has been published. These techniques include chemical exchange saturation transfer (CEST) imaging, MR fingerprinting, and simultaneous single-quantum- and triple-quantum-filtered imaging of ^23^Na (SISTINA).

**Figure 9:**
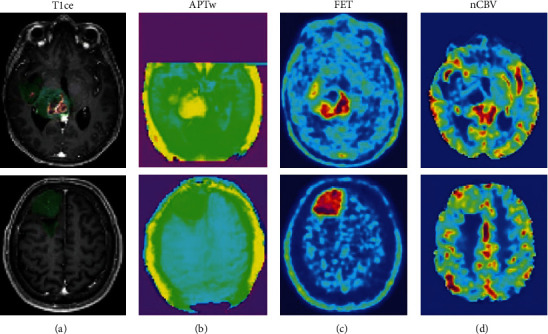
FET PET combined with amide proton transfer (APT) CEST MRI. Example images of a glioblastoma (upper row) and low-grade glioma (lower row). Segmentation (contrast-enhancing tumor (CET) in red, FLAIR hyperintense tumor (FHT) in green) is overlaid on the contrast-enhanced T1-weighted image (a) to show the spatial overlap. FHT in glioblastoma; both amide proton transfer-weighted (APTw) and CBV were significantly (*P* < 0.001, respectively) higher in PET-positive areas. Even in low-grade gliomas, a similar trend was observed for the two imaging modalities (*P* = 0.085 for APTw and *P* = 0.045 for CBV). Reproduced with permission from Figure 2 of Schon et al. [60].

CEST is a novel MRI technique in which compounds containing exchangeable protons are selectively saturated and then detected through water signal. Therefore, CEST imaging captures transfer of magnetization from mobile compounds instead of fixed compounds [66]. Amide CEST, also referred to amide proton transfer (APT), uses the amide-bound hydrogen atoms in water to image endogenous proteins. APT is emerging as a tool for cancer and ischemic stroke visualization [67], and its potential for neurooncology imaging is in its early stages of research. Two studies assessing APT CEST compared to aaPET were published during the review period. Schon et al. compared FET PET with APT imaging in 46 newly diagnosed gliomas to assess cellularity and vascularity of the tumor [60]. They found that tumor volume was larger on APT maps than FET PET and larger in GBM than in low-grade glioma. The volume of overlap between APT and FET PET images was high (median Dice score = 0.8555), as both are associated with tumor cellularity (Figure 9).

Park et al. compared MET PET to APT CEST imaging in 43 posttreatment gliomas to assess diagnostic performance [68]. They found a positive correlation between MET uptake and APT in low-grade recurrences (*r* = 0.47 and *P* < 0.001) but not high-grade recurrences, where a moderate negative correlation was reported (*r* = −0.24 and *P* < 0.001). For distinguishing recurrence in high-grade gliomas, APT (AUC 0.88) performed better than MET PET (AUC 0.71) (*P* < 0.05). These studies highlight the potential for APT CEST in neurooncology, although further research is needed to elucidate its potential compared to the other imaging modalities.

**Figure 10:**
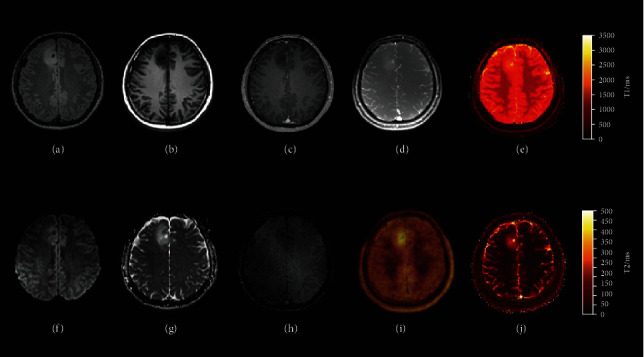
FET PET with MRI fingerprinting (MRF). Multiparametric ^18^F-FET PET-MRI and MR fingerprinting of a patient with a low-grade (WHO II) astrocytoma. (a) 3D FLAIR SPACE (sampling perfection with application optimized contrasts using different flip angle evolution) image. (b) T1-weighted MRI. (c) Postcontrast T1-weighted MRI. (d) MRF M0. (e) MRF T1-weighted image. (f) B1000. (g) ADC. (h) SWI. (i) PET/postcontrast T1-weighted MRI fusion. (j) MRF T2-weighted image. Reproduced with permission from Figure 8 of Haubold et al. [70].

MR fingerprinting is a quantitative MRI mapping technique that measures multiple tissue properties in a single acquisition, by varying MR system settings in a pseudorandom pulse sequence to generate unique signals (“fingerprints”) for each of the tissue properties of interest [69]. The demand for radiogenomics in neurooncology is increasing, and so it is important to compare MR fingerprinting with other advanced imaging modalities. Haubold et al. scanned 42 suspected primary brain tumors with FET PET and MR fingerprinting to assess their ability to predict tumor grading and mutational status, relative to histopathology as reference (Figure 10) [70]. They found that these combined modalities were able to differentiate glioma grade (AUC 0.852), ATRX mutation (AUC 0.851), MGMT mutation (AUC 0.757), IDH1 mutation (AUC 0.887), and 1p19q codeletion (AUC 0.978). These results suggest that this combination of FET PET with MR fingerprinting may prove a clinically valuable tool for noninvasive tumor molecular characterization.

Sodium MRI is a type of imaging that measures sodium concentration in tissue, using the ^23^Na ion instead of the ^1^H used in standard MR imaging [71]. The physiochemical limitations of sodium MRI include low MR sensitivity to sodium nucleus, low concentration in the body, and providing only weighted averages of intracellular signals instead of changes in concentration [72]. SISTINA is a specific sequence for sodium MRI that uses a short echo time radial projection with three-pulse triple-quantum preparation, to improve on these limitations [72]. This technique is in early stages of research, but one comparative SISTINA with aaPET study in neurooncology was published during the review period. In a pilot study, Shymanskaya et al. scanned 11 gliomas using FET PET and SISTINA sodium MRI to assess IDH mutational status [73]. The SISTINA parameters were significantly different in IDH-mutated than IDH-wt gliomas, while FET PET parameters were not predictive in this small cohort. Sodium distribution showed no spatial relation to FET uptake. Future studies in larger cohorts, and with a combination of these modalities, will be important for characterizing this potential.

## 4. Discussion

As this review illustrates, additional progress has been made in the past two years on exploring the combination and comparison of aaPET with aMRI across a range of brain tumor populations. Overall, unlike the mixed results seen in the previous older literature, a more consistent theme among most of these studies has emerged whereby aaPET outperforms aMRI for differentiating tumor characteristics such as histologic grade, tumor volume, IDH mutation, and H3K27M mutation, and for detecting infiltration, predicting and diagnosing recurrence, and predicting survival. Performance on these targets was almost universally improved by combining imaging modalities, given that they provide complementary rather than duplicative information. This highlights a critical point, that instead of considering aaPET versus aMRI, the future direction of advanced neurooncology imaging should be focused on the combined power of these imaging modalities *together*.

DWI and PWI emerge from this review as stronger aMRI techniques than MRS, which is limited by data quality issues including limited brain sampling and which is outperformed by MET and FDOPA PET. With regard to spatial correlation of aaPET and PWI, in keeping with previous reports which found mixed results, the new studies are similarly divergent and show inconsistencies in their findings. There is likely a difference in tumor type and/or PWI sequence that is causing this discrepancy. Nevertheless, it cannot be concluded that these modalities spatially align for all brain tumors. Furthermore, on volumetric analysis, aaPET tumor volume was larger, but after treatment, they tended to equalize in some studies. The previous literature also found that overall aaPET performed better than PWI, which was supported by these recent studies. Overall, both DWI and PWI are valuable imaging techniques for tumor detection, recurrence prediction, recurrence detection, and survival prediction. They were consistently outperformed on these measures by aaPET, including MET, FET, FDOPA, and AMT tracers. It will be worthwhile for researchers and clinicians to consider a dual-modality exam despite the increased overhead, given the superior performance of aaPET vs. aMRI when added to a standard CE-MRI protocol. The future potential for these modalities should be conceptualized in combination, given that they often deliver complementary information.

The emerging aMRI techniques show promise for contributing to neurooncology imaging in the future. MR fingerprinting added meaningfully to the aaPET imaging analysis. APT CEST and sodium MRI outperformed aaPET on some metrics in preliminary studies. It will be interesting to see how the research develops for these techniques, and future efforts should focus on the combinatorial benefit of using these aMRI techniques with aaPET.

### 4.1. Limitations

The advanced imaging literature reviewed here does suffer from some inherent limitations, such as asynchronous scanning in the majority of the studies, which creates potential issues of misalignment when the scans are subsequently coregistered. Ideally, PET/MRI scanning should be used but, given the limited availability of these scanners at most clinical sites, most centers can minimize time between scans. There is also the difficulty of heterogeneous populations, with studies often targeting different tumors at different time points, leading to results that are not directly comparable. Gliomas are the most represented tumor type in these studies, but attention should also be paid to imaging nonglial tumors and brain metastases. The direct comparison of studies is also complicated by differences in imaging hardware, scanner settings, acquisition protocols, imaging processing software, image processing protocols, and imaging parameter thresholds. Some studies also failed to correlate imaging findings with neuropathology results, which limits their reliability.

## 5. Conclusions

In summary, this review of the recent literature on aaPET with aMRI demonstrates additional encouraging results about the potential for these advanced imaging modalities in neurooncology. Although aaPET outperformed the aMRI techniques in the majority of the studies, there were significant gains when the modalities were combined. Specifically, DWI or PWI when combined with MET, FET, FDOPA, or AMT PET performed well for differentiating tumor grade, tumor volume, select molecular mutations, infiltration, recurrence, and predicting survival. Future efforts should continue to explore the potential for combining aaPET and aMRI in neurooncology imaging, using standardized image acquisition and processing protocols and leverage the unique and complementary information gained from each.

## Figures and Tables

**Table 1:** Summary of aaPET with DWI studies.

Study	Year	Study location	No. of patients	aaPET	DWI	Tumor type	Main results
Castello et al. [48]	2020	Rozzano, Italy	124	MET	ADC	Operated gliomas	MET PET is more sensitive for differentiating tumor grade. Both reach comparable accuracy.
Popp et al. [47]	2019	Freiburg, Germany	41	FET	ADC	Recurrent GBM	FET PET tumor volume is larger than T1w-MRI, which is larger than DWI. DWI volume only partially overlaps with FET PET and T1w-MRI, and correlated poorly with recurrence.
Lundemann et al. [49]	2019	Copenhagen, Denmark	16	FET	EPI	Pretreatment GBM	Combined parameters could map the probability of recurrence and FET PET more predictive.
Werner et al. [50]	2019	Cologne, Germany	48	FET	ADC	Pretreated high-grade glioma	FET PET is superior for differentiating tumor progression. Combination yielded highest accuracy.
Lohmeier et al. [51]	2019	Berlin, Germany	42	FET	ADC	Glioma	Both reliably differentiate recurrence from posttreatment changes, but combined provides best diagnostic accuracy.
Verburg et al. [52]	2020	Amsterdam, Netherlands	20	FET	ADC	Newly diagnosed glioma	Combination of FET PET with DWI ADC detects infiltration best in enhancing glioma, and DWI ADC with T1w-MRI is best in nonenhancing.
Piccardo et al. [54]	2019	Genoa, Italy	22	FDOPA	ADC	Pediatric DMG	Both could differentiate tumor grade, but only FDOPA PET could differentiate H3K27M-mutant status independently of histology.
Tatekawa et al. [53]	2020	Los Angeles, USA	63	FDOPA	ADC	Treatment-naïve glioma	Negatively correlated in IDH-wt and IDH-mutant-1p/19q-non-codeleted. Positively correlated in IDH-mutant-codeleted.
John et al. [17]	2019	Detroit, USA	30	AMT	ADC	Newly diagnosed GBM	Moderate correlation between AMT uptake and ADC values in nonenhancing tumor regions. Only AMT PET was associated with overall survival and recurrence.

**Table 2:** Summary of aaPET with PWI studies.

Study	Year	Study location	No. of patients	aaPET	PWI	Tumor type	Main results
Beppu et al. [56]	2019	Morioka, Japan	24	MET	ASL	Recurrent GBM	Modalities were significantly correlated before and after treatment. ASL PWI reliably predicted survival, but MET PET was more accurate.
Pala et al. [57]	2019	Günzburg, Germany	18	MET	DSC	GBM	MET PET is more sensitive than PWI DSC for residual tumor detection. PWI DSC cannot substitute MET PET in tumor detection.
Qiao et al. [58]	2019	Beijing, China	42	MET	DSC	High-grade glioma	Both could accurately differentiate radiation injury from recurrence. Combined, they yielded the best accuracy.
Roodakker et al. [39]	2019	Uppsala, Sweden	4	MET	DSC	Oligodendroglioma	MET PET could identify tumor cell density, but PWI DSC could not. The modalities were not significantly correlated.
Lundemann et al. [49]	2019	Copenhagen, Denmark	16	FET	DCE	Pretreatment GBM	FET PET had the highest predictive value for recurrence. Combined parameters could map the probability of recurrence.
Dissaux et al. [59]	2020	Brest, France	30	FET	DSC	High-grade glioma	PWI DSC is highly correlated with standard MRI, but FET PET provides complementary data.
Verburg et al. [52]	2020	Amsterdam, Netherlands	20	FET	ASL, DSC	Newly diagnosed nonenhancing glioma	FET PET with PWI DSC together could diagnose IDH-mutant glioma.
Schon et al. [60]	2020	Munich, Germany	46	FET	DSC	Newly diagnosed glioma	Both modalities could identify vascularity, but only FET PET could identify cellularity.
Fraioli et al. [61]	2020	London, UK	40	FDOPA	DSC	Glioma	Both could differentiate tumor, but performed better when combined. FDOPA PET distinguished more features.
Tatekawa et al. [53]	2020	Los Angeles, USA	61	FDOPA	DSC	Treatment-naïve glioma	Positively correlated in IDH-wt and IDH-mutant-1p/19q-non-codeleted. Not correlated in IDH-mutant-codeleted.
John et al. [62]	2019	Detroit, USA	20	AMT	DSC	GBM	Moderate correlation between modalities in nonenhancing tumor regions.

**Table 3:** Summary of aaPET with MRS studies.

Study	Year	Study location	No. of patients	aaPET	MRS	Tumor type	Main results
Kudulaiti et al. [63]	2019	Shanghai, China	109	MET	CNI	Nonenhancing supratentorial glioma	MET PET was more sensitive and specific than MRS, and when combined, sensitivity was higher and specificity stable.
Kebir et al. [64]	2019	Essen, Germany	19	MET	NAA, Cho, Cr	Newly diagnosed glioma	Both have limited potential in glioma subtyping. MET PET is better for differentiating IDH status and MRS for glioma subgrouping.
Lundemann et al. [49]	2019	Copenhagen, Denmark	16	FET	3D MRSI	Glioblastoma	FET PET had the highest predictive value for recurrence. 3D MRSI was of insufficient quality for analysis.
Verburg et al. [52]	2020	Amsterdam, Netherlands	20	FET	CNI	Newly diagnosed nonenhancing glioma	FET PET combined with other modalities detects glioma infiltration better than standard MRI or FET PET. MRS data is insufficient due to limited sampling ability.
Piccardo et al. [54]	2019	Genoa, Italy	22	FDOPA	CNI, Cho/Cr, lactate	Pediatric DMG	Both could differentiate tumor grade and H3K27M mutation, but only FDOPA PET could do so independently of histology.

Cho: choline; Cr: creatine; CNI: choline-to-N-acetylaspartate index; NAA: N-acetylaspartate.

**Table 4:** Summary of aaPET with emerging aMRI techniques studies.

Study	Year	Study location	No. of patients	aaPET	aMRI	Tumor	Main results
Schon et al. [60]	2020	Munich, Germany	46	FET	APT CEST	Newly diagnosed glioma	APT and FET PET demonstrate overlapping tumor volume for newly diagnosed glioma.
Park et al. [68]	2018	Seoul, Korea	43	MET	APT CEST	High-grade glioma	APT outperformed MET PET for differentiating recurrence.
Haubold et al. [70]	2020	Essen, Germany	42	FET	MR fingerprinting	Cerebral glioma	FET PET with MR fingerprinting could differentiate tumor grade, ATRX mutation, IDH1 mutation, and 1p19q codeletion.
Shymanskaya et al. [73]	2020	Aachen, Germany	11	FET	SISTINA	Cerebral glioma	SISTINA predicted IDH status better than FET PET.

## Data Availability

The data supporting this systematic review article are from previously reported studies included within the article and which have been cited.

## Abbreviations

aaPET: Amino acid positron emission tomography
aMRI: Advanced magnetic resonance imaging
ADC: Apparent diffusion coefficient map
AMT: ^11^C-Alpha-methyl-L-tryptophan
APT: Amide proton transfer
ASL: Arterial spin labeling sequence
AUC: Area under the curve
CE-MRI: Contrast-enhanced magnetic resonance imaging
CEST: Chemical exchange saturation transfer
Cho: Choline
CNI: Choline-to-N-acetylaspartate.
Cr: Creatine
DCE: Dynamic contrast-enhanced sequence
DMG: Diffuse midline glioma
DSC: Dynamic susceptibility contrast sequence
DWI: Diffusion-weighted imaging
FDG: ^18^F-Fluoro-deoxy-glucose
FDOPA: ^18^F-Fluoro-L-dihydroxy-phenylalanine
FET: ^18^F-Fluoroethyl-tyrosine
FLAIR: Fluid-attenuated inversion recovery
GBM: Glioblastoma
IDH: Isocitrate dehydrogenase
MET: L-[Methyl]-^11^C-methionine
MRI: Magnetic resonance imaging
MRS: Magnetic resonance spectroscopy
NAA: N-acetylaspartate
PET: Positron emission tomography
PWI: Perfusion-weighted imaging
SISTINA: Simultaneous single-quantum- and triple-quantum-filtered imaging of ^23^Na
SUV: Standardized uptake value
